# Privileged proteins with a second residence: dual targeting and conditional re‐routing of mitochondrial proteins

**DOI:** 10.1111/febs.17191

**Published:** 2024-06-10

**Authors:** Ophry Pines, Margalit Horwitz, Johannes M. Herrmann

**Affiliations:** ^1^ Microbiology and Genetics, Faculty of Medicine Hebrew University of Jerusalem Israel; ^2^ Cell Biology University of Kaiserslautern, RPTU Germany

**Keywords:** dual targeting, mitochondria, protein import, start codon, targeting signals

## Abstract

Almost all mitochondrial proteins are encoded by nuclear genes and synthesized in the cytosol as precursor proteins. Signals in the amino acid sequence of these precursors ensure their targeting and translocation into mitochondria. However, in many cases, only a certain fraction of a specific protein is transported into mitochondria, while the rest either remains in the cytosol or undergoes reverse translocation to the cytosol, and can populate other cellular compartments. This phenomenon is called dual localization which can be instigated by different mechanisms. These include alternative start or stop codons, differential transcripts, and ambiguous or competing targeting sequences. In many cases, dual localization might serve as an economic strategy to reduce the number of required genes; for example, when the same groups of enzymes are required both in mitochondria and chloroplasts or both in mitochondria and the nucleus/cytoplasm. Such cases frequently employ ambiguous targeting sequences to distribute proteins between both organelles. However, alternative localizations can also be used for signaling, for example when non‐imported precursors serve as mitophagy signals or when they represent transcription factors in the nucleus to induce the mitochondrial unfolded stress response. This review provides an overview regarding the mechanisms and the physiological consequences of dual targeting.

## Graphical abstract

The term dual localization or dual targeting refers to proteins that reside in more than one cellular compartment. Many mitochondrial proteins are dually localized and populate, in addition to mitochondria, the cytosol, the nucleoplasm, chloroplasts, and other compartments. The review article introduces prominent examples of dually localized mitochondrial proteins and describes the different molecular mechanisms that can lead to the dual targeting of mitochondrial proteins.

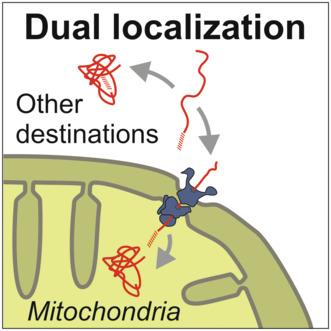

**Abbreviations**
EMC: ER membrane complex
ER: endoplasmic reticulum
ERAD: ER‐associated degradation
IMS: intermembrane space
MDV: mitochondria‐derived vesicle
MTS: matrix‐targeting signal
NLS: nuclear localization signal
SRP: signal recognition particle
TA: tail anchor

## Introduction

The function of a eukaryotic cell relies on its proteome. Cellular protein biosynthesis is an intricate process as thousands of different proteins need to be synthesized in the cytosol, targeted to their respective intracellular location and folded into their final conformation. Problems in the folding and assembly of proteins can lead to neurodegeneration and aging disorders [1, 2]. Thus, the (patho)physiology of misfolded and aggregated proteins is a central problem in molecular cell biology. However, the reliable targeting of proteins to their respective locations and the subsequent translocation into these compartments might be similarly error‐prone and comparably important for cellular function. Therefore, mistargeted, similar to misfolded, proteins are under surveillance of quality control systems that either degrade these proteins or sequester them in protein aggregates [3, 4, 5, 6]. While targeting of proteins to specific subcellular compartments appears to be precise, imperfect “sloppy” targeting of proteins can occur under stress. On the other hand, such “imperfection” may be intentional and a mechanism of targeting proteins to multiple compartments.

The identity of cellular compartments depends on their specific protein repertoire. The reliable and efficient targeting of proteins to their correct locations is therefore essential [7, 8, 9]. Targeting signals on proteins serve as address labels for specific compartments. N‐terminal signal sequences on proteins that are targeted to the endoplasmic reticulum (ER) are a prime example for targeting sequences. Their recognition by the signal recognition particle (SRP) stalls protein synthesis to ensure a highly reliable translocation through the Sec61 translocon of the ER membrane [10, 11]. If ER targeting and translocation fails, the same signal sequences are recognized by quality control factors to induce rapid degradation [3].

The situation is often less black‐and‐white for other compartments. In the case of the nucleus, for example, many proteins that display a nuclear targeting signal (NLS) show some degree of distribution between the nucleus and the cytosol and, hence, the proteome of the nucleoplasm and the cytosol show a considerable overlap [12]. Worth emphasizing, in this case, is that import into the nucleus occurs through aqueous pores and does not include translocation through membrane bilayers such as in the ER, mitochondria, and peroxisomes.

What is the situation for mitochondrial proteins: black‐and‐white or somewhat gray? Initially, it was assumed that an N‐terminal matrix‐targeting sequence (MTS), ensures absolute translocation into mitochondria [13]. For many proteins, this might be the case; however, for other presequence‐containing mitochondrial precursors that accumulate in the cytosol, some are efficiently recognized in the cytosol by quality control factors and degraded [14, 15, 16]. Alternatively, non‐imported precursor proteins can be sequestered in the cytosol in specific protein aggregates, termed MitoStores [17, 18], or they associate with other cellular compartments [19, 20, 21, 22]. A completely different situation is the intentional dual targeting of mitochondrial proteins which are stable in mitochondria and the cytosol, a frequent and important phenomenon [20, 23, 24, 25, 26]. Dual targeting can be the consequence of different and fascinating molecular mechanisms. This review article intends to provide an overview of these mechanisms and the physiological implications of protein dual targeting.

## Mechanisms of protein dual targeting

Numerous examples of dually localized proteins have been described in the past and, in many cases, the underlying mechanisms of some have been fully deciphered. Rather than providing long lists of proteins and mechanisms, we will describe characteristic and, if possible, physiologically important examples of dually localized proteins. The focus will be on proteins of human and *Saccharomyces cerevisiae* (from here on referred to as yeast) cells, owing to the importance of the organism and the comprehensive knowledge about the yeast model. We will refer to targeting mechanisms, physiological relevance, and the regulation that controls intracellular protein distribution. Interestingly, the dual targeting mechanism of a certain protein may be different in different organisms. For example, fumarase (fumarate hydratase) is dual targeted by two genes in *Arabidopsis thaliana*, by one mRNA with two translation initiation sites in rat, by multiple mRNAs in human and by a single translation product in yeast [27].

### Multiple genes

**Fig. 1:**
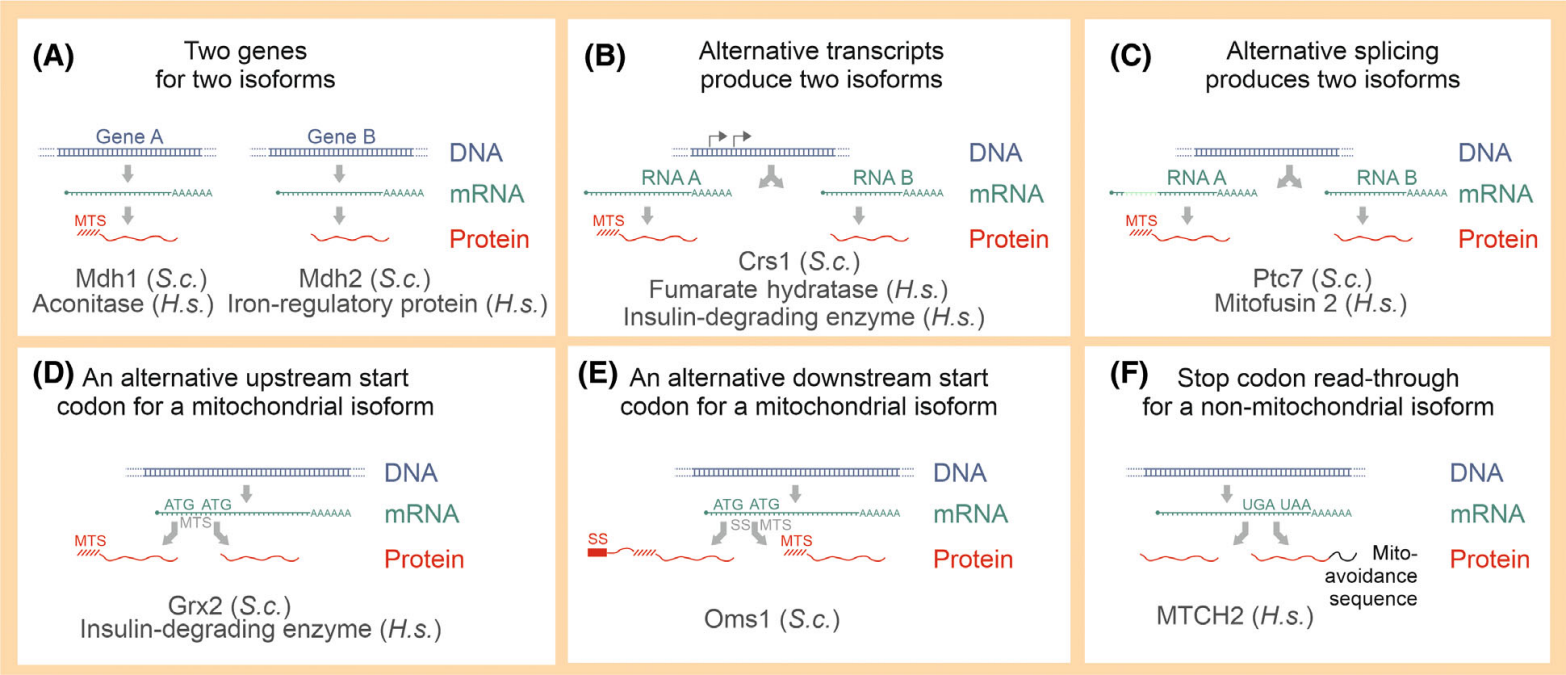
Different mechanisms can lead to dually localized protein isoforms. These different mechanisms are sketched here in simplified versions and well‐characterized examples from human (*H*.*s*., *Homo sapiens*) or yeast (*S*.*c*., *Saccharomyces cerevisiae*) are indicated. (A) Two isoforms can be expressed from distinct genes. (B, C) One gene can give rise to different mRNAs which produce protein isoforms that are targeted to different cellular locations depending on their inclusion of alternative start codons or alternative splicing. (D–F) Alternative start (ATG) or stop (UGA, UAA) codons lead to different translation products. Particularly, the use of upstream start codons for the generation of a mitochondrial isoform is frequent. The preference of translational initiation for the alternative start codons can differ considerably, influencing the balance of the mitochondrial versus non‐mitochondrial isoforms. MTS, matrix‐targeting signal; ss, signal sequence for protein targeting to the endoplasmic reticulum.

Different molecular mechanisms lead to the synthesis of dually localized protein isoforms (see Fig. 1 for an overview). In many cases, cells express distinct isoforms from separate gene loci (Fig. 1A). If gene duplication occurred rather recently, the sequence homology can be very high, but in other cases, the gene duplications happened early in eukaryotic evolution and thus, the dually targeted isoforms are found throughout eukaryotes. In many cases, enzymes have isoforms in the cytosol and the mitochondrial matrix, such as for malate dehydrogenase in yeast, where the Mdh1 isoform serves as an enzyme in the mitochondrial citrate cycle and the closely related Mdh2 isoform promotes gluconeogenesis in the cytosol [28]. The levels of both proteins are tightly regulated, Mdh1 mainly at the level of expression and Mdh2 also by catabolite‐dependent degradation [29, 30]. Interestingly, Mdh2 is additionally translocated into peroxisomes by a piggyback transport mechanism [31]. Mdh1 and Mdh2 are distinct proteins, synthesized from different genes and their regulation is not mutually influenced by the other isoform. Comparable isoforms of malate dehydrogenases are also found in human cells, where the cytosolic isoform (malic enzyme) assists the malate–aspartate shuttle that transfers reducing equivalents from the cytosol to mitochondria [32].

Aconitase is another enzyme of the citrate cycle found in two functionally distinct, but structurally related isoforms. In addition to the canonical enzyme of the matrix, the enzyme is present in the cytosol and nucleoplasm of human cells and contains specialized aconitase isoforms, called iron regulatory proteins which are involved in the control of iron metabolism by binding mRNA to repress translation or degradation [33, 34]. The iron–sulfur clusters of this protein are rather labile and the loss of one iron from the cluster is used for sensing iron‐limited growth conditions. Thus, here the two isoforms have very different physiological roles, still both are essential for life in animals. Worth mentioning, aconitase is also dual targeted in yeast but by a different mechanism and it participates in the glyoxylate shunt in the cytosol [35, 36], thereby demonstrating that the evolutionary escapade of localization and function can be remarkable.

### Distinct transcripts

From a genetics perspective, a particularly interesting scenario involves a single gene which gives rise to two or more distinct protein isoforms. Different transcripts can be produced from different transcriptional start sites (Fig. 1B). For example, the yeast genome contains only one gene for cysteinyl‐tRNA synthetase (Crs1) that gives rise to two transcripts, a longer transcript encoding the mitochondrial isoform and a shorter one for the cytosolic protein [37]. If respiration is induced, increased amounts of the longer isoform are produced to support mitochondrial protein synthesis.

A similar mechanism is used by the human gene for fumarase (fumarate hydratase) which produces multiple transcripts. Again, the longer transcripts contain an additional in‐frame start codon for synthesis of the presequence‐containing mitochondrial isoform [38]. The shorter variant is targeted to the nucleus where it plays a role in the DNA damage response [39, 40, 41]. Intriguingly, upon DNA damage in both human and yeast cells, fumarase moves into the nucleus where it brings about the accumulation of fumarate, which in turn affects factors of the DNA damage repair system [27]. This function in the DNA damage response is apparently evolutionary very ancient and is even found in bacteria [42, 43]. Thus, fumarase is an excellent example of how eukaryotic cells use dual localization for metabolic regulation.

Alternative splicing serves as another mechanism to produce dually localized isoforms (Fig. 1C). This situation is common in mammalian cells and numerous examples have been described. For example, the mitochondrial fusion factor mitofusin 2 (MFN2) is encoded by a gene that also generates alternatively spliced transcripts resulting on the production of proteins ERMIT2 and ERMIN2 (for ER mitofusin 2 tether and ER mitofusin 2, respectively). These isoforms are inserted into the ER membrane and form contact sites with the outer membrane proteins MFN1 and MFN2 via extended coiled‐coil interactions [44, 45]. Thus, alternative splicing is used to create two halves of a molecular bridge between the ER and mitochondria. Each of the two bridge components is targeted to a different compartment by specific sequences encoded by the different exons.

The gene for the insulin‐degrading enzyme is another example for the synthesis of a splice variant that encodes a mitochondrial precursor [46]. This mitochondrial isoform, together with the structurally related presequence peptidase PreP [47], facilitates the degradation of processed presequences [48, 49].

Since most yeast genes lack introns, alternative splicing is uncommon in yeast. However, there are a few examples of transcripts, for which the retention of a non‐functional intron is used to produce an alternative translation product. For example, the spliced transcript for the phosphatase Ptc7 produces a mitochondrial precursor, whereas the non‐spliced variant is localized to the nuclear envelope by virtue of a transmembrane domain encoded in the exon sequence [50]. Protein dephosphorylation by the mitochondrial Ptc7 isoform stimulates the activity of several mitochondrial proteins, including enzymes of the citrate cycle, and the alternative splicing of Ptc7 presumably has a regulatory role, yet the full molecular details remain to be determined [51].

### Alternative translation products

Arguably, the most common mechanism to produce dually localized isoforms is the use of alternative start codons (Fig. 1D). One single study in human cells alone identified 126 different examples [52] and presumably many more exist. Likewise, in yeast a very large number of cases were identified in which non‐canonical translation initiation generates a cryptic pool of mitochondrial proteins [53]. Thus, codons such as CTG, TTG, GTG, ACG, ATC, ATA, and ATT upstream of the canonical open reading frames serve as minor start sites that generate longer translation products with an N‐terminal MTS. Mitochondrial localization was verified for several examples including Adh4, Trr1, Trz1 and Hyr1 [53].

*Vice versa*, established mitochondrial proteins can contain down‐stream start sites that give rise to shorter isoforms lacking mitochondrial presequences. Indeed, methionine residues are frequently present in many mitochondrial precursors and positioned right after the mitochondrial presequence peptidase cleavage site. *In vitro*, these methionine codons give rise to shorter translation products (known as pseudo‐mature forms in the community) [54, 55]. Whether *in vivo* these ATG codons always also serve as minor translation start sites is unknown but a considerable number of characterized cases have been reported. For example, the yeast glutaredoxin Grx2 is synthesized as two isoforms, the longer one containing an N‐terminal MTS that is generated by an upstream start site [56]. These isoforms control the redox states of a distinct sets of proteins that reside in the cytosol or in the matrix [57]. Interestingly, a very small fraction of the short (cytosolic) Grx2 isoform resides in the mitochondrial intermembrane space (IMS) to prevent the glutathionylation of Mia40 [58] and other IMS proteins; overexpressing this IMS‐Grx2 species is toxic as it counteracts Mia40‐dependent protein oxidation [59]. Thus, the design of the Grx2 sequence is well suited to direct large amounts of this reducing enzyme to the cytosol and matrix, and small (yet important) amounts to the mitochondrial IMS. Other redox enzymes, including the glutathione reductase Glr1, employ the same elegant distribution mechanism [60].

The generation of sulfur from cysteine is an essential process in human and yeast cells, catalyzed by the enzyme NifS (called Nfs1 in yeast). This enzyme is required in mitochondria (for the biogenesis of iron–sulfur cluster) and in the nucleus (for the production of sulfur‐containing RNA modifications). In human cells, the protein is made from two start codons giving rise to a longer mitochondrial and a shorter nuclear isoform [61]. Which start codon is preferred can be influenced by the specific conditions and the enzyme activity is apparently dynamically distributed according to the prevailing needs. In yeast, the nuclear isoform of Nsf1 is produced from an incompletely translocated import intermediate that reverse translocates back into the cytosol [62].

In most cases in which alternative start sites are used, the longer isoform represents the mitochondrial precursor. The yeast fumarase reductase Osm1 is an exception to this pattern [63]. The longer isoform of Osm1 is made with a signal sequence for ER targeting (Fig. 1E) whereas the shorter form is directed into the IMS of mitochondria to serve as an electron acceptor for Mia40‐mediated protein oxidation under anaerobic conditions [64].

In addition to alternative start sites, the conditional readthrough of stop codons can be used to generate dually targeted isoforms (Fig. 1F). This mechanism can influence the targeting of proteins with C‐terminal signals, such as in the case of tail‐anchored proteins of the outer membrane. For the outer membrane protein MTCH2, a C‐terminally extended version was described where the addition sequence avoids mitochondrial targeting [65]. MTCH2 serves as an insertase that integrates tail anchor proteins into the outer membrane, but the function of non‐mitochondrial isoforms is unknown [66, 67].

## Dual targeting of single translation products

### Folding of cytosolic precursors

**Fig. 2:**
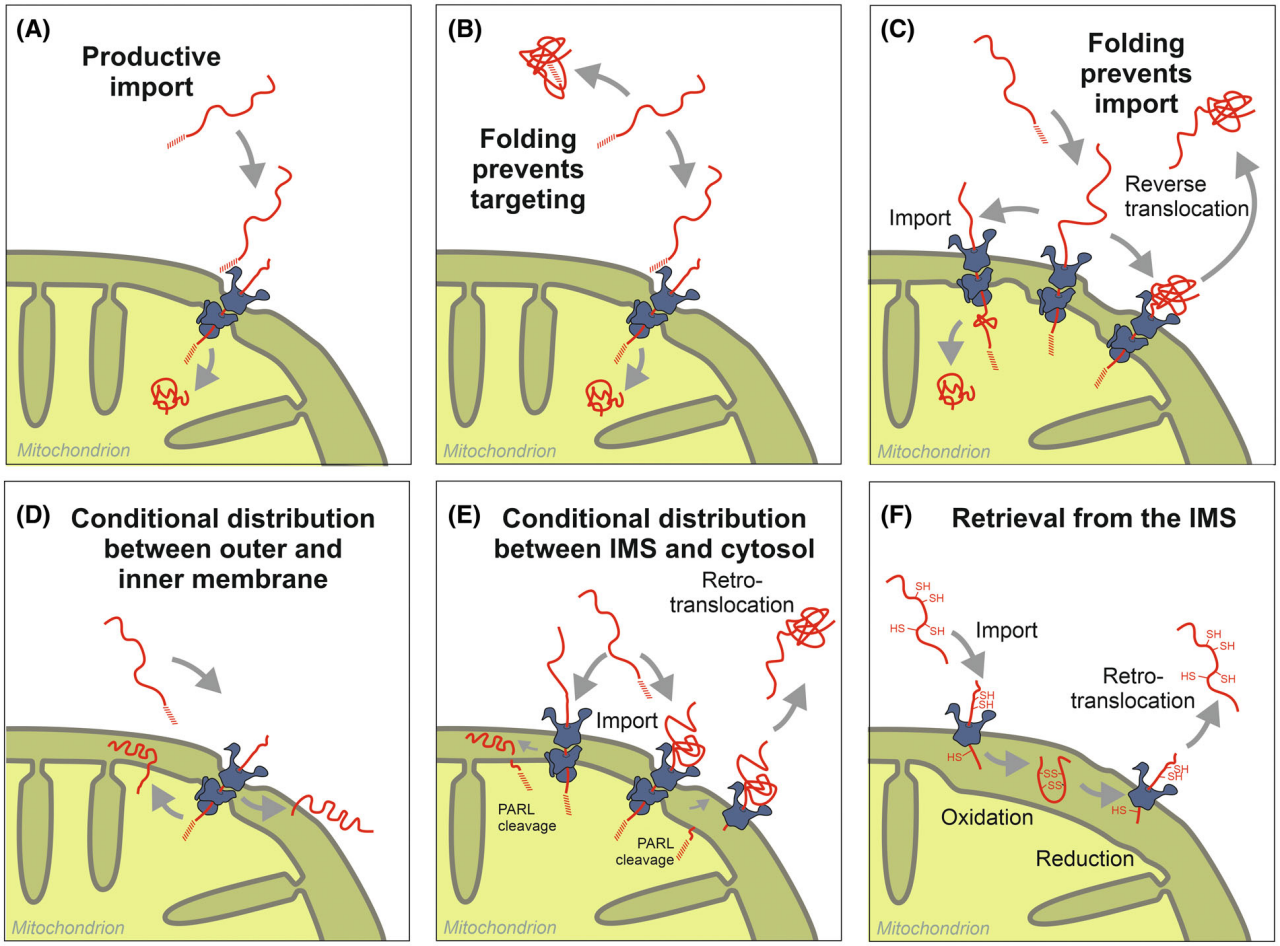
Folding of precursors in the mitochondria and the cytosol can lead to the dual targeting of isoforms. (A) Efficient mitochondrial targeting is presumably the typical situation for most precursors. (B) Folding can prevent the import into mitochondria for example by masking the matrix‐targeting signal (MTS) in the structure of the folded proteins. Alternatively, the assembly with other proteins or interaction with membranes might impair protein import. (C) The folding of C‐terminal regions of proteins can lead to reverse translocation. The driving force for forward productive import is folding of the protein in the matrix, whereas the driving force for reverse translocation is folding of the protein C terminus in cytosol. The latter is a mature protein from which the MTS was removed. (D) Membrane proteins can be distributed to the outer and inner membrane. These proteins often have poor targeting signals and their final location is influenced by the energetic state of the mitochondria. (E) Cleavage of an import intermediate by the rhomboid protease PARL in the inner membrane leads to the dual distribution of the lipid transfer protein STARD7 in the IMS and the cytosol. (F) Proteins with structural disulfide (‐SS‐) bonds can be retro‐translocated from the IMS to the cytosol under reducing conditions which open their disulfide bonds converting them to reduced thiols (‐SH).

Up to this point, we have discussed the production of distinct translation products which are targeted to different intracellular locations. Nevertheless, a single protein population can be distributed between two or more subcellular compartments (see Fig. 2 for overview). Since only unfolded proteins are efficiently targeted to mitochondria (Fig. 2A), folding (and assembly) can irreversibly lock precursors in the cytosol (Fig. 2B). The accumulation of non‐processed precursors in the cytosol have been observed for different cases, but molecular details remain often unclear.

More frequent is “reverse translocation” in which a fraction of a given protein is not fully imported into mitochondria but rather translocation intermediates move back into the cytosol owing to the folding of their C‐terminal domain (Fig. 2C). In such cases, the extra‐mitochondrial species typically accumulates in its proteolytically matured form. Indeed, in most cases that were analyzed, such cytosolic (or nuclear) species represented mature proteins. The best‐characterized example for a folding‐induced reverse translocation into the cytosol is the yeast form of fumarase which was the first report of dual targeting of a single translation product [68, 69]. In the case of yeast fumarase, a translocation intermediate can either be fully translocated into the mitochondrial matrix or reverse‐translocated back into the cytosol. The driving force for both translocation or reverse translocation is folding of the precursor; if the N terminus of the precursor starts to fold in the matrix it will be fully imported but if the C‐terminal folds first, the protein will be blocked for forward movement and will reverse translocate back to the cytosol. Accordingly, mutations throughout the fumarase sequence, which impair its folding, the overexpression or depletion of either mitochondrial or cytosolic chaperones as well as the efficiency of the mitochondrial import machinery affect fumarase distribution [22, 70, 71, 72].

Several studies reported the presence of the mitochondrial chaperonin subunits Hsp10 and Hsp60 in the cytosol of human cells, particularly in tumor cells [73, 74, 75, 76]. In yeast, Hsp60 is exclusively mitochondrial, however, the expression of cytosolic version of Hsp60 increases under stress of yeast cells, compatible with the hypothesis that the cytosolic fractions found in human cancer cells might have a protective function [77]. Worth pointing out is that dual targeting can be affected by processes related to protein folding such as interaction of the protein with other proteins or the chemical modification of the polypeptide chain [78, 79].

Intraorganellar folding can also regulate the distribution of proteins between the IMS and the matrix. In the case of the complex I biogenesis, factor NDUFAF8 dual localization is achieved by a two‐step import pathway in which the protein is first forms an intermediate in the IMS that further translocates into the matrix. Dual distribution between the IMS and the matrix depends on the transfer rates across the outer and inner membrane and is influenced by the folding of the NDUFAF8 intermediate in the IMS [80].

The mechanism referred to above is mechanistically reminiscent of a number of proteins that distribute between the outer and inner membrane of mitochondria (Fig. 2D). The by far best‐characterized example is PINK1, a central regulator of mitophagy and an important factor in the context of Parkinson's disease [81, 82]. In healthy, well‐energized mitochondria, PINK1 is targeted to the inner membrane where it is rapidly degraded [83, 84, 85]. Degradation of PINK1 is mediated by the rhomboid protease PARL and the i‐AAA protease YME1L, which are part of a large super‐complex in the inner membrane that is organized by the prohibitin‐like protein SLP2 [86]. However, in compromised mitochondria, a fraction of PINK1 remains in the outer membrane and serves as a mitophagy signal to trigger the removal of compromised mitochondria. The details of this dual targeting of PINK1 and the (patho)physiological consequences of this phenomenon were covered in detail in many excellent reviews [87] and will therefore not be discussed here in depth. However, this example shows how dual targeting can be used in signaling: in this case, the outer membrane‐targeted PINK1 serves as an eat‐me signal in compromised human mitochondria.

The first protein that was shown to participate between the inner and outer membrane, long before PINK1 was discovered, was the yeast cytochrome b_5_ reductase Mcr1. The Mcr1 precursor has a weak MTS followed by a hydrophobic transmembrane domain. Since the poor signal directs only a fraction of the protein to the TIM23 complex in the inner membrane, the residual fraction accumulates at the level of the outer membrane where it is integrated into the lipid bilayer [88]. Weak targeting signals are a reiterating feature of many dually localized proteins.

The lipid transfer protein STARD7 is a particularly interesting case of a dually localized protein for which the molecular mechanisms were recently described and which lead to the distribution of the protein between the IMS and the cytosol [89, 90]. STARD7 forms an import intermediate this is cleaved by the rhomboid protease of the inner membrane, PARL. Upon PARL cleavage the protein can either remain in the IMS or retro‐translocate back into the cytosol (Fig. 2E). Both species are physiologically relevant as they coordinate the levels of coenzyme Q in mitochondria and the plasma membrane. The cytosolic species thereby shuttles coenzyme Q to the plasma membrane and protects cells against ferroptosis [90].

Retro‐translocation from the IMS has been described as another mechanism of dual targeting of mitochondrial proteins [91]. Many IMS proteins contain cysteine residues that are critical for their import into mitochondria. The oxidation of these cysteine by the oxidoreductase Mia40 traps these proteins in the IMS [92, 93, 94, 95, 96]. Reducing conditions can open the disulfide bonds and induce the release of Mia40 substrates back into the cytosol [91] in a process called retro‐translocation (Fig. 2F). These proteins are rather stable in the cytosol and give rise to dually localized isoforms that populate the IMS and the cytosol [97, 98].

## Dual targeting as a consequence of ambiguous signals

**Fig. 3:**
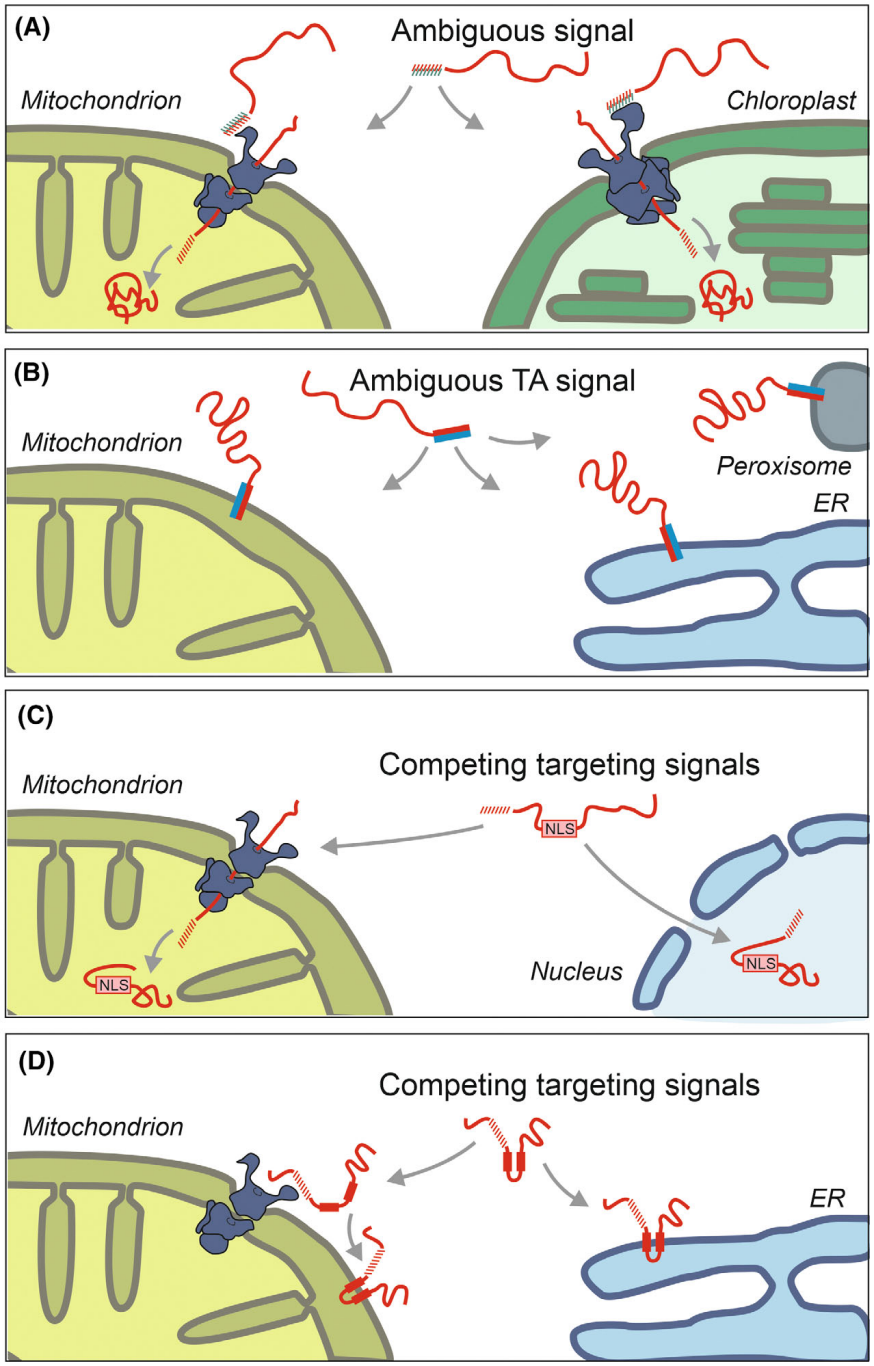
Proteins can be distributed between different compartments as a consequence of ambiguous or competitive targeting signals. (A) In plant cells, proteins are localized to both mitochondria and chloroplasts since their targeting signals have characteristics of matrix‐targeting signals (MTSs) and chloroplast transit peptides. (B) Tail anchor (TA) sequences are C‐terminal hydrophobic stretches that integrate into different membranes. Insertases facilitate membrane integration, however, the insertion is not highly specific and the same TA proteins can be found in different compartments, including mitochondria, peroxisomes and the endoplasmic reticulum (ER). (C) Mitochondrial precursors contain targeting signals for the nucleus (NLS, nuclear localization signal). When there is reduction in mitochondrial import efficiency, these proteins are directed to the nucleus. (D) The mitochondrial protein contains an internal MTS that facilitates its targeting to the mitochondrial outer membrane as the predominant species, however, it is also recognized by the signal recognition particle (SRP) and inserted into the ER membrane.

Many mitochondrial proteins of plant cells have weak and ambiguous MTSs that also function as a chloroplast targeting signal (or transit peptide) [99] (Fig. 3A). As a consequence, the proteomes of mitochondria and chloroplast considerably overlap [100]. Transit peptides and mitochondrial presequences share similar features despite some characteristic elements. Both are of helical structure, rich in positively charged and hydroxylated residues and have one positively charged and one hydrophobic surface. However, transit peptides are often longer and more complex, containing proline residues and hydrophobic segments [101, 102, 103]. These specific features serve as sorting codes that determine which fraction of each protein ends up in mitochondria versus chloroplasts [104, 105]. Post‐translational modifications of the targeting sequences can further modulate the organelle specificity in plants [106, 107]. Thus, chloroplast‐containing cells employ dual targeting as an economic and wide‐spread mechanism to distribute hundreds of proteins between their two distinct endosymbiotic organelles [108].

The intracellular distribution of proteins with tail anchor (TA) sequences is another example where ambiguous signals are used to distribute proteins between different destinations (Fig. 3B). TA sequences are C‐terminal transmembrane domains that anchor proteins to the cytosol‐exposed side of organelles. The hydrophobicity and the short sequence of residues following the transmembrane domain serve as targeting information. Many ER proteins contain highly hydrophobic TA sequences whereas a number of positive charges in the very C‐terminal hydrophilic tail of the proteins include mitochondrial targeting information [109, 110]. Most TA proteins do not show a black‐and‐white distribution to one specific compartment but populate different membranes to different degrees [111]. Their individual distribution is influenced by the specificity of the insertases that integrate them into target membranes and by quality control factors that extract mistargeted TA proteins and pass them on to their appropriate membranes [112, 113, 114, 115, 116, 117, 118, 119]. In human cells, the outer membrane protein MTCH2 mediates the insertion of mitochondrial proteins [66], and these mitochondrial proteins are actively repelled by a selectivity filter that is present in the ER membrane complex (EMC) that inserts ER proteins [111, 120, 121, 122].

## Dual targeting as consequence of competing signals

A systematic screen in yeast identified nuclear targeting signals (NLS) in a large number of mitochondrial precursor proteins [20]. These proteins accumulate in the nucleus only if the mitochondrial import system is compromised or overwhelmed. Thus, such proteins are *bona fide* mitochondrial proteins under benign conditions but wind up in the nucleoplasm upon mitochondrial dysfunction (Fig. 3C). Such a conditional dual localization is best known from Atfs1, a transcription factor of *Caenorhabditis elegans*, which induces the mitochondrial unfolded protein stress response UPRmt [123]. Atfs1 is targeted to mitochondria by its MTS under non‐stress conditions, but when the function of mitochondria and protein import are compromised, Atfs1 is transported into the nucleus, there it induces the expression of mitochondrial chaperones and other stress‐responsive proteins. Atfs1‐induced gene expression is also important to allow cells to recover from starvation, as it facilitates the replication of the mitochondrial DNA and the expansion of the mitochondrial network [124, 125]. The human transcription factor ATF5 presumably reacts to mitochondrial stress conditions by a similar mechanism [126]. However, the human induction of UPRmt is more complex and involves other factors, some of which, such as MNRR1, are also induced by the conditional dual localization to the nucleus [127, 128]. Particularly interesting is the case of the mitochondrial protein DELE1 which, upon stress conditions, is cleaved by the mitochondrial protease OMA1 to give rise to a C‐terminal fragment that retro‐translocates into the cytosol to trigger a stress‐response pathway [129, 130].

In yeast, a competition of targeting signals was also found for the ubiquitin ligase Ubx2 which is predominantly found in the ER, but also present in the mitochondrial outer membrane (Fig. 3D). This factor promotes the ubiquitination of precursors that arrest in the mitochondrial import pore [131]. Dual localization was also reported for other ER quality factors suggesting that the ER‐associated degradation (ERAD) and the mitochondria‐associated degradation share critical components [16, 30, 132, 133].

## Novel dual targeting mechanisms of mitochondrial proteins

### Mitochondria‐derived vesicles

**Fig. 4:**
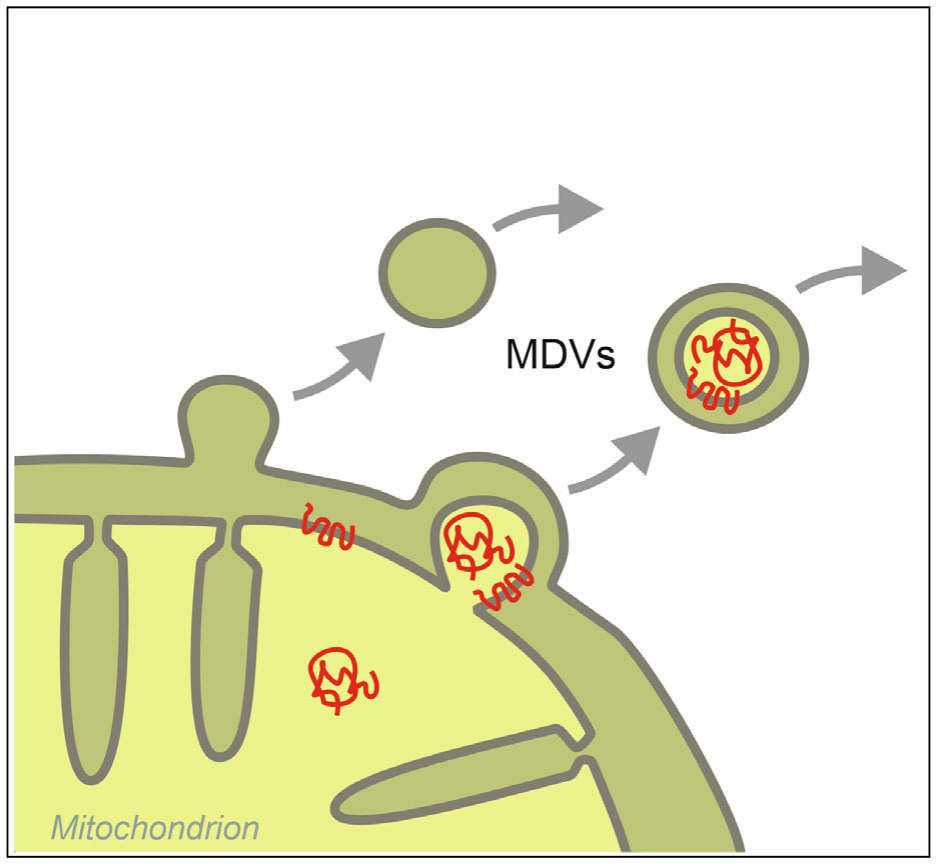
Novel mechanisms of dual targeting. Mitochondria can give rise to mitochondria‐derived vesicles (MDVs) which can lead to the export of mitochondrial proteins to other locations in the cell. These mitochondrial isoforms can accumulate as stable factors that function inside or outside the cell.

For ERAD, luminal ER proteins are exported to the cytosol. A comparable export of mitochondrial proteins from the matrix has not been shown. However, mitochondria might still send proteins to other cellular destinations via Mitochondrial Derived Vesicles (MDVs, in yeast also called mitochondria‐derived compartments). These single or double membrane‐enclosed vesicles are formed and released from mitochondria in response to various triggers (Fig. 4) [134, 135]. MDVs interact with other organelles such as lysosomes and peroxisomes or may be incorporated and excreted via extracellular vesicles [136, 137]. MDVs selectively incorporate specific protein and lipid cargoes and are involved in various functions such as (a) mitochondrial quality control, (b) immunomodulation, (c) energy complementation and (d) compartmentalization and transport [138, 139]. A good example are MDVs from yeast that carry selective protein cargo enriched for ATP synthase subunits [140]. Remarkably, these MDVs harbor a functional ATP synthase complex, have a membrane potential, and can produce ATP. These vesicles may fuse with naive mitochondria to regenerate ATP‐deficient mitochondria and may participate in organelle‐to‐organelle communication.

## Final remark

When the mechanisms of intracellular protein targeting were identified in the 80s and 90s, it was assumed that each protein finds its specific location by use of an unambiguous address label. This one‐protein‐one‐destination pattern is certainly wrong in many cases. In yeast, technologies based on cross‐complementing enzyme fragments, split‐GFP reporters, proteomics of cellular fractions or proximity labeling showed that the subcellular proteome is not black‐and‐white; the intracellular distribution of each protein might rather show its specific shade of gray. Since in most cases, the minor isoforms are outshined by their predominant species, a phenomenon termed “eclipsed distribution” [141], their function is often obscured. This is particularly true for human cells, which are much bigger and harbor a complex and larger proteome, express proteins from multiple splice variants, differentiate into different tissue types, and employ multifaceted stress response programs. This leads to a much greater heterogeneity, and the full extent of dual targeting in human cells is waiting to be explored in the future.

## Conflict of interest

The authors declare no conflict of interest.

## Author contributions

All authors wrote the paper. JMH prepared the figures.
